# Immune Checkpoints OX40 and OX40L in Small-Cell Lung Cancer: Predict Prognosis and Modulate Immune Microenvironment

**DOI:** 10.3389/fonc.2021.713853

**Published:** 2021-11-25

**Authors:** Peixin Chen, Hao Wang, Lishu Zhao, Haoyue Guo, Liping Zhang, Wei Zhang, Chenglong Sun, Sha Zhao, Wei Li, Jun Zhu, Jia Yu, Chunyan Wu, Yayi He

**Affiliations:** ^1^ School of Medicine, Tongji University, Shanghai, China; ^2^ Department of Medical Oncology, Shanghai Pulmonary Hospital, School of Medicine, Tongji University, Shanghai, China; ^3^ Department of Pathology, Shanghai Pulmonary Hospital, Tongji University School of Medicine, Shanghai, China; ^4^ Anhui No. 2 Provincial People’s Hospital, Hefei, China

**Keywords:** small cell lung cancer, OX40 (CD134), OX40 ligand, tumor immune microenvironment, prognosis

## Abstract

**Background:**

OX40 and OX40 ligand (OX40L), as essential immune checkpoint (IC) modulators, are highly correlated with cancer immunity regulation as well as tumor microenvironment (TME). Immunotherapy showed outstanding advantages in small-cell lung cancer (SCLC) therapy. However, functions and clinical significance of OX40 and OX40L in SCLC were not clear yet.

**Materials and Methods:**

SCLC samples of 143 patients were collected for immunohistochemistry (IHC) or whole-exome sequencing (WES). We comprehensively explored the expression and mutation of OX40/OX40L in SCLC, and systematically linked OX40/OX40L with TME.

**Results:**

The expression of OX40/OX40L on tumor cells and tumor-infiltrating lymphocytes (TILs) was found in the IHC cohort and verified in other cohorts with SCLC tissues and cell lines. The results showed co-expression patterns among OX40/OX40L, other ICs, and T-cell markers. The WES data suggested that OX40/OX40L mutation is rare in SCLC (<5%). Patients with positive OX40 protein expression on TILs showed substantially higher recurrence-free survival than those with negative expression (p=0.009). The external dataset also indicated that high OX40/OX40L expression was correlated with better prognosis [overall survival: OX40, p<0.001; OX40L, p=0.019]. Importantly, activation of immunity and high infiltration of CD4(+) and CD8(+) T cells were observed in the high OX40/OX40L expression group.

**Conclusions:**

Collectively, this work highlighted the significance of OX40 and OX40L in prognosis and TME cell infiltration characterization of SCLC. Evaluating the OX40/OX40L-expression levels of individual patients with SCLC might contribute to guiding more precise therapy.

## Introduction

According to the latest cancer statistics, lung cancer remained the primary causes of death of oncology patients all over the world (1–3). There is a growing trend in the incidence of non-small-cell lung cancer (NSCLC) and small-cell lung cancer (SCLC) in recent years (1–3). SCLC, which comprised between 10 and 15% of total lung cancer, is a rapidly proliferating and highly aggressive tumor (1–4). For extensive disease SCLC (ED-SCLC), platinum-based doublet chemotherapy was the standard therapy. Despite the initially high response rate (approximately 60%), a large proportion of patients with SCLC would relapse within 2 years (5–8). Drug resistance severely affected the prognosis of SCLC patients. Immunotherapy, such as programmed cell death ligands-1 (PD-L1) inhibitor and programmed cell death-1 (PD-1) inhibitor, was considered as a major breakthrough in tumor treatment. The clinical benefits of immune checkpoint (IC) inhibitors in SCLC were revealed by several clinical trials (9–13). Clinical data suggested that the response rate of immunotherapy was higher than that of chemotherapy alone (9–13). Both CASPIAN and IMPOWER 133, two randomized phase 3 trials, highlighted the application prospect of PD-L1 inhibitor in first-line treatment of ED-SCLC (9, 10). However, the first-line immunotherapy plus platinum–etoposide merely extended overall survival (OS) to 12.3–13 months from a previous 10.3 months of the chemotherapy group (9, 10). These clinical studies also found the unsatisfying predictive performance of PD-L1 expression in immunotherapy efficacy and prognosis (9, 10). CheckMate 311, another phase 3 trial of immunotherapy *versus* standard chemotherapy in SCLC, failed in achieving statistically significant difference in OS (14). Thus, exploring the clinical values of other immune-related markers in SCLC might be an effective method to introduce novel combined immunotherapy and develop useful prognostic biomarkers.

OX40 and OX40 ligand (OX40L), serving as promising target of tumor immunotherapy, were expressed on various types of cancers and immune cells (15–22). The interaction of OX40 and OX40L promoted the stimulation and accumulation of T cells, resulting in the antitumor effect (15, 16). The conflicting prognostic implications of OX40/OX40L was found in different types of cancers. In NSCLC, melanoma, and colorectal cancer, OX40/OX40L indicated improved prognosis (17–19). The high proportion of OX40(+) cells inhibited distant metastasis in malignant melanoma (19). However, in another cohort with early-stage NSCLC, high OX40 expression was correlated with early recurrence and shorter OS (20). Similar negative roles of OX40/OX40L in prognosis were also found in liver cancer and leukemia (21, 22). In SCLC, the amplification of OX40, also known as tumor necrosis factor receptor superfamily member 4 (TNFRSF4), was found (23). For SCLC cases, the expression profile of OX40 and OX40L and their roles in clinical outcome and tumor microenvironment (TME) remained unclear. The limited clinical benefits of immunotherapy in SCLC might ascribe to the finite cognition of tumor immune microenvironment (24–26). For these reasons, we identified the OX40/OX40L protein expression and gene mutation in SCLC by immunohistochemistry (IHC) and whole-exome sequencing (WES). Then, the relationship among OX40, OX40L, PD-1, PD-L1, CD3, CD4, CD8, FOXP3, tumor mutation burden (TMB), OS, recurrence-free survival (RFS), and other clinicopathological characteristics was fully explored. We determined the signal pathways and immune cell infiltration features of patients with SCLC on the basis of OX40/OX40L expression. Above clinical findings were verified in addition cohorts.

## Patients and Methods

### Patients’ Enrollment and Sample Collection

From 2014 to 2019, a total of 143 eligible patients with SCLC were enrolled in the study. We gathered tumor tissues and blood samples of patients with SCLC before any clinical therapy. We collected and reviewed complete electronic records of all enrolled cases. The SCLC staging were determined by the tumor-node-metastasis (TNM) system. With the approval of the ethics committee of Shanghai Pulmonary Hospital, China (ethical numbers: K20-022), we conducted the study. All participants signed informed consents at the start of study.

### IHC and Cutoff Value for OX40/OX40L

We measured the expression of OX40 and OX40L in 102 SCLC specimens by means of IHC. Under the strict asepsis procedure, samples were acquired and diagnosed as SCLC by pathology. After formalin fixation and paraffin embedment, pathology slides were made and prepared for further staining. We used the routine method of dewaxing. Then, specimens were soaked in citrate buffer for antigen recovery. The usage of 3% hydrogen peroxide helped block the activity of endogenous peroxidase. Later, fetal bovine serum was used as blocking reagent. Primary antibodies (OX40, 61637, Rabbit mAb, Cell Signaling Technology; OX40L, ab211287, Rabbit mAb, Abcam) and secondary antibody were applied standardly. In the end, the commonly used diaminobenzidine colorimetry and digital microscope (IX73, OLYMPUS) were applied to quantify protein expression levels of OX40 and OX40L. Three microscope fields at 20× magnification was selected randomly for expression evaluation. The expression of OX40 and OX40L on cancer cells and tumor-infiltrating lymphocytes (TILs) was scored by two pathologists independently.

The survival analysis was the approach to determine the best cutoff point of OX40 and OX40L (27). According to survival analysis results, the p value of the best cutoff value was the minimum. For OX40, more than 20% staining on cancer cells was defined as positive expression, while any staining on TILs was deemed positive expression. The scope of positive OX40L expression on tumor cells (TCs) and TILs was more than 80% staining and 10% staining, respectively.

### DNA Extraction and Sequencing

We performed WES on 41 SCLC cases. After the standard process of sample collection, the DNA characteristics of SCLC tissues and blood samples were analyzed by WES. For DNA extraction and quantification, we used QIAamp DNA Tissue Kit (Qiagen, Valencia, USA), RelaxGene Blood DNA system (Tiangen, Beijing, China), Fluorometer (Qubit 2.0), and the Qubit dsDNA HS assay kit (Thermo Fisher Scientific, CA, USA). Once we obtained fragmented genomic DNA by particular instrument and reagents (28, 29), we constructed DNA library by Kapa Biossystems (MA, USA). Then, fragments with poor quality were excluded, while remaining reads were amplified by a certain circulation of polymerase chain reaction. The Illumina Novaseq 6000 platform was used for DNA sequencing.

With the help of Genome Analysis ToolKit (V 4.1) and Mutect2, DNA fragments were aligned to hg19 reference genome (GRch37), thus detecting somatic mutation, single nucleotide variants, and insertion-deletion mutations. Eventually, according to conventional formula for TMB calculation (28), the somatic TMB value of each case was obtained.

### Public Datasets Acquisition and Processing

We aimed at further investigating the clinical significance of OX40 and OX40L in the SCLC public cohorts. We queried the Cancer Genome Atlas (TCGA) Database (https://portal.gdc.cancer.gov) and the Gene Expression Omnibus (GEO) Database (https://www.ncbi.nlm.nih.gov/geo/) to get datasets that match the inclusive criteria. All enrolled datasets must own complete RNA sequencing (RNA-seq) and clinicopathologic data for human SCLC specimens. In addition, we surfed a website, named the Cancer Cell Line Encyclopedia (CCLE, https://portals.broadinstitute.org/ccle) Database (30) to verify the expression of OX40 and OX40L in SCLC cell lines. R Studio software (V4.0.1) and several R packages were installed for exporting data from public databases and conducting subsequent bioinformatics analysis, such as differently expressed genes (DEGs) identification, functional analysis, etc.

### Gene Biological Role and Function Analysis

The biological functions and pathway enrichment of DEGs between two groups were explored by the Gene Ontology (GO) analysis (http://www.geneontology.org/) and the Kyoto Encyclopedia of Genes and Genomes (KEGG) pathway analysis (https://www.kegg.jp/). The GO bar charts consisted of three sections, namely, cellular components, biological processes, and molecular functions. The KEGG plot demonstrated significantly DEGs-related pathways.

### Estimation of TME Characterization in SCLC

In the current study, we conducted the Estimation of STromal and Immune cells in MAlignant Tumours using Expression data (ESTIMATE) algorithm and CIBERSORTx algorithm on the global gene expression profiles of SCLC patients. The ESTIMATE algorithm provided the overall abundance of immune cells and stromal cells of each clinical sample (31). Apart from the immune score and the stromal score, the tumor purity of each SCLC case was also deduced by the ESTIMATE algorithm (31). As one of deep deconvolution and machine-learning tools, the CIBERSORTx used the linear support vector regression and a set of leukocyte genes called LM22 (32, 33). For CIBERSORTx algorithm, it can statistically infer the particular infiltration percentage of 22 kinds of TILs in SCCL TME, such as subgroups of T cells, B cells, natural killer (NK) cells, etc.

### Statistical Analysis

The chi-square tests and Pearson correlation tests were used for detecting relationship among OX40/OX40L, PD-1, PD-L1, other biomarkers, and clinical factors. In addition, the Mann-Whitney U test was applied to compare continuous variables between two groups. For survival analysis, the Kaplan-Meier approach and the log-rank test were utilized. By means of univariate and multivariate logistic regression method, factors that affected the expression status of OX40 and OX40L were found. Through univariate and multivariate Cox regression method, independent prognostic factors in SCLC were also identified. Two-sided p value less than 0.05 was deemed to be statistically significant. Two statistical tools were used in the research, namely, the SPSS software (V 22.0) and the RStudio software (V4.0.1).

## Results

### Clinical and Tumor Features of Patients

Totally, the whole cohort contained 143 patients with SCLC (**Table 1** and **Table S1**). The age of participants ranged from 38 to 84 years old. A high percentage of patients were males (121/143, 84.6%) and smokers (77/143, 53.8%). The baseline characteristics of the IHC group and the WES group were separately detailed in **Tables S2** and **S3**. The sample size of the IHC cohort and the WES cohort was 102 and 41, respectively.

**Table 1:** Clinical and tumor characteristics of the whole cohort (n=143).

Variables	No. (%)	Variables	No. (%)
Sex		T stage	
Female	22 (15.4)	T1–2	97 (67.9)
Male	121 (84.6)	T3–4	46 (32.1)
Age, median, years	65	N stage	
<70	110 (76.9)	N0–1	71 (49.7)
≥70	33 (23.1)	N2–3	72 (50.3)
Smoking history		Metastasis	
Non-smoker	66 (46.2)	No	120 (83.9)
Smoker	77 (53.8)	Yes	23 (16.1)
SCLC TNM staging			
I–II	61 (42.7)		
III–IV	82 (57.3)		

N, lymph node; SCLC, small-cell lung cancer; T, tumor, TNM, tumor-node-metastasis.

### Expression Characteristics of OX40 and OX40L in SCLC

**Figure 1:**
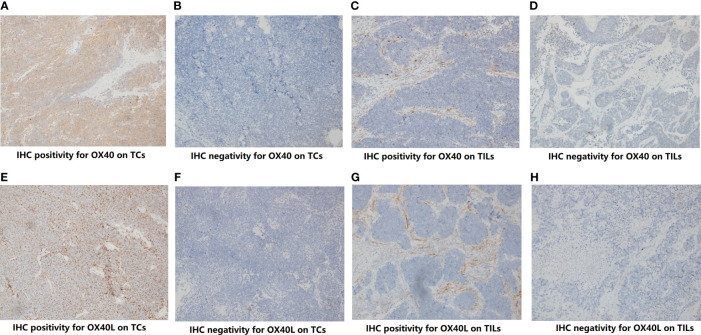
The protein expression of OX40 and OX40 ligand (OX40L) on tumor cells (TCs) and Infiltrating lypmphocytes (TILs). **(A)** IHC positivity for OX40 on TCs. **(B)** IHC negativity for OX40 on TCs. **(C)** IHC positivity for OX40 on TILs. **(D)** IHC negativity for OX40 on TILs. **(E)** IHC positivity for OX40L on TCs. **(F)** IHC negativity for OX40L on TCs. **(G)** IHC positivity for OX40L on TILs. **(H)** IHC negativity for OX40L on TILs.

Through IHC, the protein expressions of OX40 and OX40L were found on both TCs and TILs (**Figure 1**). For OX40, the positive rate on cancer cells and TILs was 7.8% (8/102) and 72.5% (74/102), respectively. Of these 102 tissues, 22 samples exhibited positive OX40L staining on TILs (21.6%), while only two samples showed positive OX40L staining on TCs (2.0%). We then explored the correlation among OX40, OX40L, clinical factors, and eight conventional IHC markers (**Table S4**). The OX40 protein expression status on TILs had contacts with TNM staging (p=0.044), synaptophysin (p=0.009), and P40 (p=0.013). However, no significant relationship was found between TCs’ OX40 expression and clinical factors. On TILs, the expression degree of OX40 was not significantly linked to enrolled binary markers.

In the study, we also investigated relevance between OX40/OX40L and immune markers (**Figure S1A** and **Table S5**). The expression level of OX40 on TILs was significantly correlated with OX40L expression on TILs (p=0.048), PD-1 expression on TILs (p=0.003), PD-L1 on TILs (p=0.019), CD3 (p<0.001), CD4 (p=0.001), and CD8 (p=0.023). OX40L on TILs also had widespread connection with PD-1, PD-L1, and several major markers of T cells (both p<0.05). There was a distinct correlation between OX40 and OX40L expression on TCs (p=0.001). Except for OX40L on TCs, the negative correlation between malignant cells’ OX40 expression and other markers was indicated.

The genetic expressions of OX40 and OX40L in SCLC were verified in 22 SCLC human samples that were extracted from GSE43346 and 54 cell lines that were collected in the public CCLE Database (**Figure S1**). As shown in **Figure S1B**, mRNA expressions of OX40 and OX40L in SCLC samples were higher than them in normal tissues. When compared with NSCLC cell lines, the relative low expression levels of OX40 and OX40L in SCLC cell lines were displayed (**Figure S1C**).

### Logistic Regression Analysis of OX40 and OX40L Expression

The logistic regression analysis was used to study factors predicting expression of OX40 and OX40L in SCLC. Regarding the limited number of cases that showed positive OX40 or OX40L expression on TCs, we separately developed the logistic regression models for OX40 and OX40L on TILs (**Tables S6**, **S7**). We calculated odds ratio (OR) and 95% confidence interval (CI) of each factor for quantitative analysis. On TILs, eight variables were identified as underlying predictors in OX40 expression status by univariate logistic regression analysis, while none of these was deemed to be significantly predictive factor by multivariate logistic regression analysis (**Table S6**). Similar negative results were found in OX40L expression (**Table S7**).

### Survival Analysis for OX40 and OX40L in SCLC

**Figure 2:**
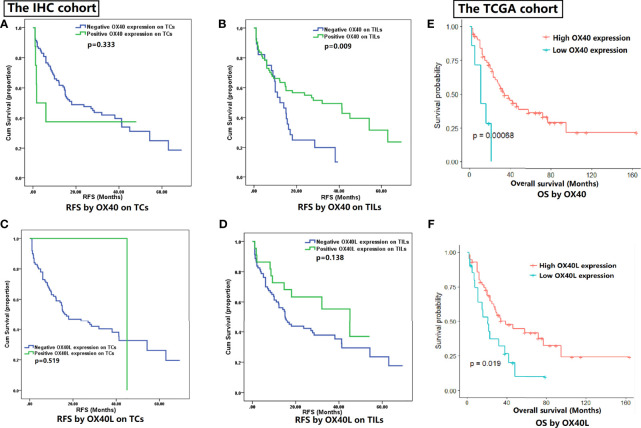
Survival analysis for OX40 and OX40 ligand (OX40L) expression. **(A)** RFS by OX40 on TCs. **(B)** RFS by OX40 on TILs. **(C)** RFS by OX40L on TCs. **(D)** RFS by OX40L on TILs. **(E)** OS by OX40. **(F)** OS by OX40L.

RFS data of 102 patients with SCLC was collected in the IHC cohort. Higher recurrence rate was found in patients with smoking history (31/44, 70.5%) when compared with non-smokers (33/58, 56.9%). The Kaplan-Meier curves reflected prognostic differences between different expression status groups (**Figure 2**). On TCs, OX40 expression status had no marked relation with RFS (p=0.333, **Figure 2A**). The overexpression of OX40 on TILs significantly elevated clinical profits in SCLC (vs low OX40 expression: RFS 34.7 months, 95% CI 27.8-41.5 vs 16.3 months, 95% CI 11.5-21.1, p=0.009; **Figure 2B**). Regretfully, there was no statistical significance in the survival analysis of OX40L in SCLC (**Figures 2C, D**).

On the basis of OX40 and OX40L expression in SCLC, we conducted the subgroup analysis in the primary cohort (**Figure S2**). We combined OX40 and OX40L with each other and tested their influences on outcomes (**Figure S2**). TILs’ OX40 in combination with TCs’ OX40L (p=0.015) or TILs’ OX40L (p=0.012) could effectively distinguish patients with different prognosis.

Given the meaningful findings in the IHC cohort, we further verified the clinical values of OX40 and OX40L in the public TCGA cohort (**Figures 2E, F**). The OS data of 77 SCLC patients in the TCGA cohort were downloaded online (34). The survival analysis demonstrated OS was better in SCLC patients with higher expression levels of OX40 (p<0.001) and OX40L (p=0.019), which had high consistency with our IHC results.

### Cox Regression for Survival Analysis

Sixteen clinicopathological traits were enrolled in the univariate and multivariate Cox regression analyses for RFS (**Table 2**). All cox regression outcomes were analyzed by using hazard ratio (HR) and 95% CI. By means of univariate Cox regression analysis, nine potential prognostic indicators were found, including age (HR=1.744, p=0.045), smoking status (HR=1.612, p=0.064), TNM staging (HR=1.854, p=0.014), OX40 on TILs (HR=0.502, p=0.011), PD-L1 on TILs (HR=0.604, p=0.068), CD3 (HR=0.613, p=0.052), CD4 (HR=0.627, p=0.089), FOXP3 (HR=0.564, p=0.049) and CD8 (HR=0.577, p=0.058). Then, we performed multivariable regression analysis on the above nine factors. After adjustment of confounding characteristics, multivariate results suggested that smoking status (HR=1.915, p=0.029) and OX40 on TILs (HR=0.523, p=0.032) were significant prediction factors in patients with SCLC.

**Table 2:** Cox regression analysis for recurrence-free survival in the whole IHC cohort*.

Variables	Univariate			Multivariate		
	HR	95% CI	p	HR	95% CI	p
**Gender (Female *vs.* Male)**	1.373	0.678–2.280	0.379			
**Age (<70 *vs.* ≥70)**	1.744	1.014–3.000	**0.045**	1.448	0.816–2.571	0.206
**Smoking status (Non-smoker *vs.* Smoker)**	1.612	0.973–2.669	**0.064**	1.915	1.070–3.430	**0.029**
**SCLC staging (I–II *vs.* III)**	1.854	1.130–3.040	**0.014**	1.488	0.898–2.467	0.123
**OX40 on TCs (negative *vs.* positive)**	1.560	0.624–3.903	0.342			
**OX40 on TILs (negative *vs.* positive)**	0.502	0.296–0.851	**0.011**	0.523	0.290–0.944	**0.032**
**OX40L on TCs (negative *vs.* positive)**	0.529	0.073–3.828	0.528			
**OX40L on TILs (negative *vs.* positive)**	0.604	0.307–1.190	0.145			
**PD-1 on TILs (negative *vs.* positive)**	0.799	0.479–1.332	0.390			
**PD-L1 on TCs (negative *vs.* positive)**	1.373	0.430–4.388	0.593			
**PD-L1 on TILs (negative *vs.* positive)**	0.604	0.352–1.037	**0.068**	0.958	0.424–2.161	0.917
**CD3 (negative *vs.* positive)**	0.613	0.374–1.005	**0.052**	0.742	0.357–1.540	0.423
**CD4 (negative *vs.* positive)**	0.627	0.366–1.073	**0.089**	1.095	0.514–2.333	0.815
**CD8 (negative *vs.* positive)**	0.577	0.327–1.018	**0.058**	1.076	0.4712–2.457	0.863
**FOXP3 (negative *vs.* positive)**	0.564	0.319–0.997	**0.049**	0.688	0.262–1.705	0.399

HR, hazard ratio; SCLC, small-cell lung cancer; TCs, tumor cells; TILs, tumor-infiltrating lymphocytes; PD-1, program death-1; PD-L1, program death-ligand 1; FOXP3, forkhead box protein P3; OX40L, OX40 ligand; P, P value for whole; 95% CI, 95% confidence interval. Statistically significant data were marked with bold and underline. *Recurrence-free survival data was updated in May, 2021.

### Mutational Status of OX40/OX40L and Their Associations With Prognosis

The statistics analysis implied that the mutation frequency of OX40 was 4.9% (2/41), while OX40L did not show any mutation in SCLC tissues. Concurrent mutations were found in OX40 and 14 genes, including ADGRB3, BBX, FRMPD3, LAMA5, MUC16, MUC5B, OR5L2, OTOG, PCDH10, PGP, TAAR8, THSD4, and TP53. Then, the somatic TMB values of 41 SCLC samples were calculated for further analysis. As shown in **Figure S3A**, the OX40 mutation group represented higher TMB values (*vs.* the OX40 wild-type group: TMB 7.545 *vs.* 7.214), but the difference was not significant (p>0.05).

In the whole WES cohort, the progression-free survival (PFS) information was collected in 36 patients (87.8%). In addition, the OS data were obtained in 31 subjects (75.6%). In both PFS and OS, instead of having a perfect separation between two survival curves, the Kaplan-Meier curve of OX40 mutation cases met the curve of OX40 wild-type cases, indicating few influences of OX40 mutation status on prognoses of patients with SCLC (**Figures S3B, C**).

### GO and KEGG Enrichment Analyses for OX40 and OX40L in SCLC

**Figure 3:**
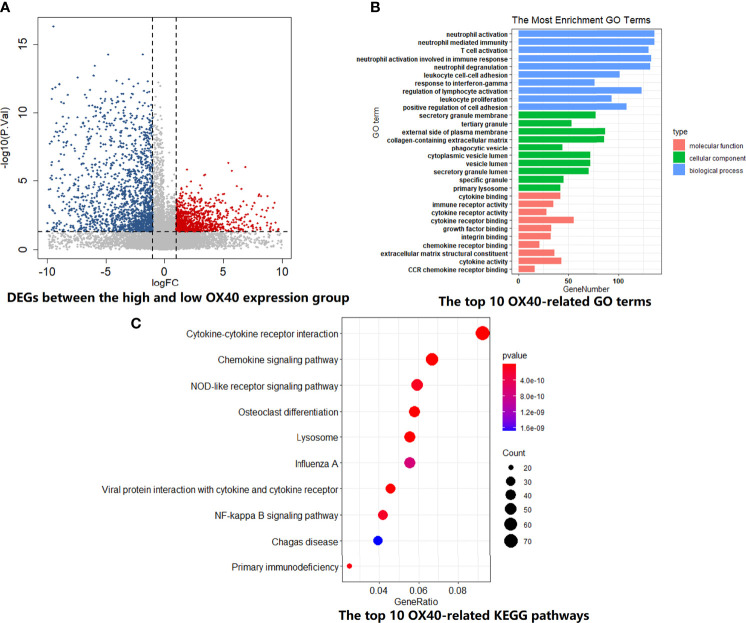
GO and KEGG enrichment analysis for OX40 in SCLC. **(A)** Differentially expressed genes (DEGs) between the high and low OX40 expression groups were visualized in the volcano plot. **(B)** The top I0 OX40-relatd GO terms in the field of cellular component, biological process, and molecular function. **(C)** The top I0 OX40-relatd KEGG pathways. GO, Gene Ontology; KEGG, Kyoto Encyclopedia of Genes and Genomes; SCLC, small cell lung cancer.

In order to explore the biological behaviors between different OX40 and OX40L expression patterns, we performed GO and KEGG enrichment analyses on the TCGA dataset. For OX40, the high-expression group contained 41 subjects, while the rest of the subjects were allocated to the low-expression group (n=40). Of all DEGs between the two groups, a large proportion of DEGs downregulated in the high-OX40-expression group (2,719/4,405, 61.7%; **Figure 3A**). As shown in **Figures 3B, C**, high OX40 expression was markedly enriched in activation pathways of immune cells, such as neutrophil activation (GO:0042119, p=2.16E-39), T cell activation (GO:0042110, p=5.92E-39), leukocyte proliferation (GO:0070661, p=4.50E-32), and cytokine–cytokine receptor interaction (hsa04060, p=5.66E-15). Concrete top 10 OX40-related functions, biological processes, and pathways are summarized (**Tables S8**, **S9**).

**Figure 4:**
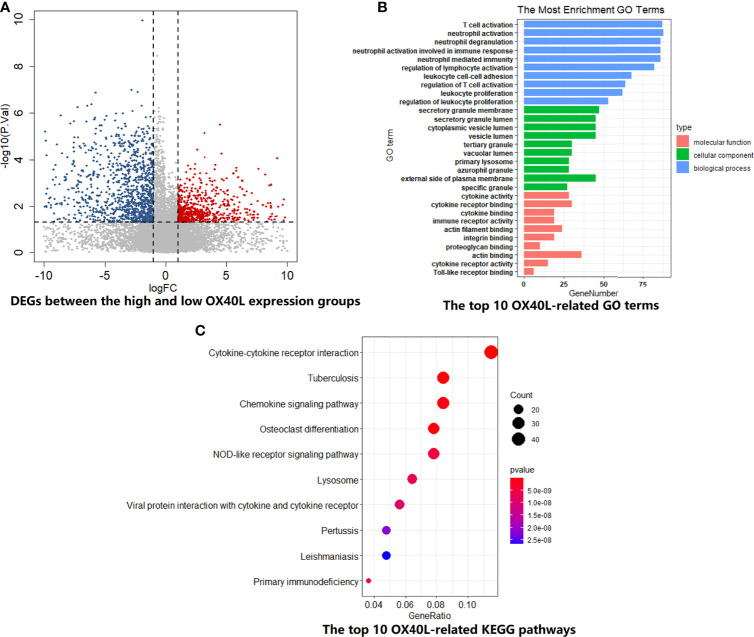
The biological functions and pathway enrichment of OX40L-related DEGs in SCLC. **(A)** A total of 3048 DEGs were identified between the high and low OX40L expression group. **(B)** The GO enrichment results of OX40L-related DEGs in SCLC. Go terms were divided into three types, including cellular component, biological process, and molecular function. **(C)** The KEGG enrichment results of OX40L-related DEGs in SCLC. DEGs, differentially expressed genes; GO, Gene Ontology; KEGG, Kyoto Encyclopedia of Genes and Genomes; SCLC, small cell lung cancer; OX40L, OX40 ligand.

For OX40L, similar grouping method was adopted. The volcano plot demonstrated the enrichment or depletion of 3,048 DEGs in SCLC patients with high OX40L expression level (**Figure 4A**). Specifically, the ratio of downregulated genes to upregulated genes was closed to 2:1 (1,941:1,107). As the ligand of OX40, OX40L also principally related to immune-related functions and pathways. There was a huge overlap between the GO and KEGG enrichment analyses results of OX40 and OX40L in SCLC (**Figures 4B, C** and **Tables S10**, **S11**). Therefore, we hypothesized that OX40 along with OX40L might have important functions in antitumor immunity of SCLC.

### TME Features Mediated by OX40/OX40L in SCLC

**Figure 5:**
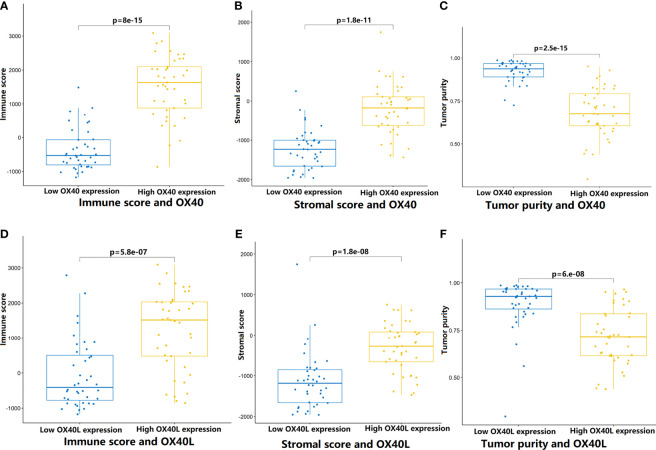
Comparison of tumor microenvironment features that mediated by OX40/0X40L in SCLC. **(A–C)** By the ESTIMATE algorithm, the differences of immune score, stromal score, and tumor purity between the high and low OX40 expression groups. **(D–F)** Comparison of immune score, stromal score, and tumor purity between the high and low OX40L expression groups. SCLC, small cell lung cancer; OX40L, OX40 ligand.

**Figure 6:**
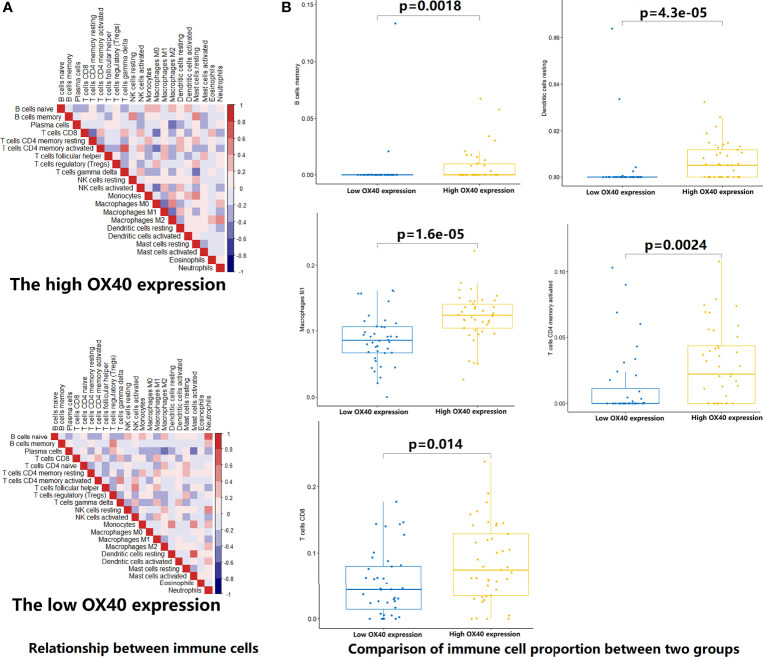
Estimation of relative infiltration proportions of 22 kinds of immune cells in the high and low OX40 expression group. **(A)** By the CIBERSORTx algorithm, relationship matrixes of 22 immune cells in small cell lung cancer patients wit h high and low OX40 expression. **(B)** The high OX40 expression group presented increased infiltration of 5 kinds of immune cells.

**Figure 7:**
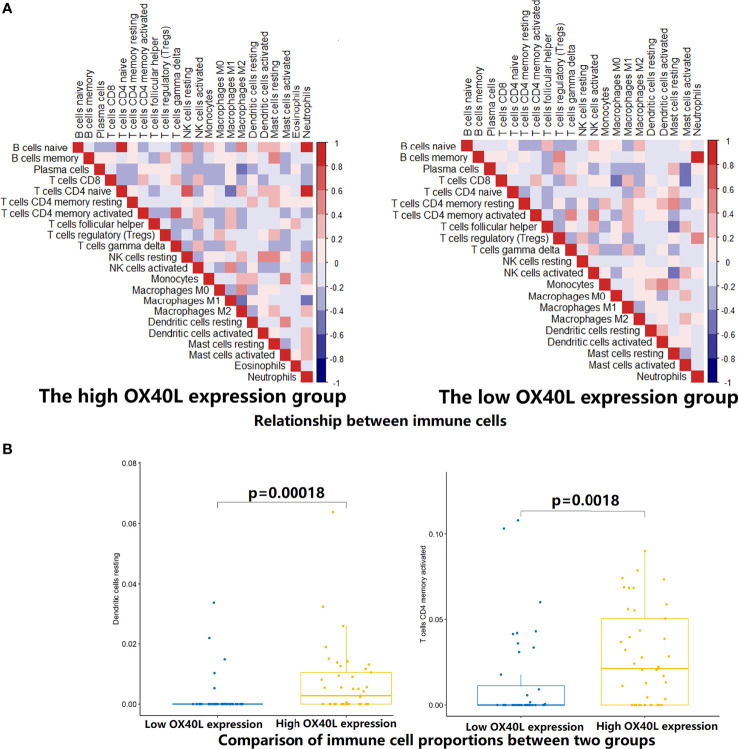
Estimation of relative infiltration proportions of 22 kinds of immune cells in the high and low OX40 ligand (OX40L) expression group. **(A)** By the CIBERSORTx algorithm, relationship matrixes of 22 immune cells in small cell lung cancer patients with high and low OX40L expression. **(B)** The infiltration of resting dendritic cells (p=O.OOO18) and activated memory CD4+ T cells (p=0.0018) was elevated in the hi OX40L group.

To test the hypothesis, we compared the TME landscapes between the high- and low-expression groups by the ESTIMATE and CIBERSORTx methods (**Figures 5** **–** **7**). The overall abundance of immune cells and stromal cells was higher in the high-OX40- or high-OX40L-expression group (**Figure 5**). Relative low tumor purity was found in tissues with high OX40 or OX40L expression. Moreover, the CIBERSORTx results were consistent with above findings and further confirmed our hypothesis (**Figures 6**, **7**). The TME with high OX40 expression significantly existed increased immune infiltration of various types of immune cells, including memory B cells (p=0.0018), resting dendritic cells (p=4.3e-05), M1 macrophages (p=1.6e-05), activated memory CD4+ T cells (p=0.0024), and CD8+ T cells (p=0.014; **Figure 6**). For the high-OX40L-expression group, the enrichment of TME resting dendritic cells (p=0.00018) and activated memory CD4+ T cells (p=0.0018) infiltration was also found (**Figure 7**). To sum up, OX40 and OX40L acted as vital immunoregulators in SCLC.

### Development of the OX40/OX40L-Based Immune Interaction Network

For the sake of illustrating distinct expression patterns among OX40, OX40L, and immune genes (IGs), we developed the OX40/OX40L-based immune network. The gene set of 2,518 IGs was available in the ImmPort Database (35). The correlation analysis was firstly implemented to quantify the relationship between OX40/OX40L and 1,347 IGs, which were tested in the public TCGA cohort. Correlation coefficients and p value were calculated. The number of significantly OX40-related IGs and OX40L-related IGs was 471 and 360, respectively. A small portion of gene expression levels were inversely associated with OX40 expression (25/471, 5.3%). Then, 50 significantly OX40/OX40L-related IGs with correlation coefficient more than 0.70 were selected for subsequently analysis. For survival analysis, subjects of the TCGA cohort were split evenly on the basis of expression level of target gene. Eventually, eight IGs with the p value equal to or less than 0.1 in the survival analysis were chosen for the construction of the OX40/OX40L-based immune interaction network (**Figure S4**). The interactions among OX40, OX40L, NCR3, CSF1, TGFB1, RELB, TNFRSF8, HCST, TNFRSF1B, and CARD11 were extensive (**Figure S5**).

## Discussion

The whole analysis of the current study centered on the exploration of roles of OX40 and OX40L in SCLC. We comprehensively investigated the expression levels and mutation frequencies of OX40 and OX40L in patients with SCLC. Our statistical analysis indicated that OX40 and OX40L were closely associated with clinical characteristics, prognosis, TMB, immune-related genes and pathways, and TME immune cell infiltration characterization. Although the functions of OX40 and OX40L were reported in various cancers, including NSCLC, this is, to our knowledge, the first study to fully investigate the clinical and biological significances of OX40 and OX40L in SCLC.

OX40 is a type 1 glycoprotein, while OX40L is a type II glycoprotein (36). The expressions of OX40 and OX40L were detected in various kinds of tumor tissues, such as NSCLC and oral carcinoma (17, 20, 37, 38). In SCLC, we found that TILs’ OX40 expression was associated with TNM staging, and TCs’ OX40L expression was related to age, indicating different expression patterns of OX40 and OX40L in different cancer types. The mutation of TNFRSF4, encoding OX40, led to the deficiency of T cells, thus inducing Kaposi sarcoma, an endothelial malignancy (39, 40). Additionally, for TNFSF4, which encode OX40L, its mutation downregulated the risk of atherosclerosis and myocardial infarction (41). TNFRSF4 and TNFSF4 mutations in cancers were rarely reported. Similarly, our current research also revealed that most SCLC samples failed to find TNFRSF4 or TNFSF4 mutation.

As crucial ICs, OX40 and OX40L exhibited giant prospects in clinical application. High OX40/OX40L expression was correlated to better clinical prognosis in patients with NSCLC, melanoma, and colorectal cancer (17–19, 42, 43). Nevertheless, high OX40/OX40L also suggested poor prognosis in hepatocellular carcinoma, acute leukemia, as well as head and neck squamous cell carcinoma (21, 22, 44, 45). Given the contradictory results of prognostic effect of OX40/OX40L in cancers, it is worth evaluating the influences of OX40 and OX40L on prognosis of SCLC patients. By survival analysis in the local and public cohorts, we found that SCLC patients with high OX40L expression showed superior prognosis than those with low OX40L expression. Moreover, SCLC patients with positive OX40 expression on TILs significantly relapsed later and lived longer. Similar meaningful result of OX40 in prognosis was obtained by bioinformatics analysis. In glioblastoma, Ichiyo Shibahara et al. revealed that the immunoregulation effect of OX40 principally changed with the TME (46). This might explain why OX40/OX40L showed different influences on prognosis in various cancers.

Mounting evidence displayed that OX40-OX40L signals have an indispensable role in immune cell activation and antitumor immunity. In NSCLC, there was a robust correlation between OX40 expression on TILs and FOXP3 (17), which was consistent with our findings in the SCLC cohort. By IHC, we found that FOXP3 could predict OX40 expression status on TILs. The mechanisms of OX40/OX40L in regulating FOXP3 expression and Treg expansion were complex. Several studies showed that the dynamic equilibrium of Treg cells partly rely on OX40/OX40L axis (47–51). Accumulating researches highlighted the immunomodulation effects of OX40 and OX40L in T cells (52–54). OX40/OX40L mediated the differentiation of helper T cell (Th) through NF-kappa B pathway (55). OX40/OX40L axis also enhanced activity and effects in T cell subsets through PI3K/PKB, MAPK, and NFA T pathways (52–54). In SCLC, the top 10 OX40/OX40L-related biological processes and pathways were also enriched in the activation, maturation, and proliferation of T cells, such as cytokine–cytokine receptor interaction, chemokine signaling pathway, NF-kappa B signaling pathway, etc. Positive correlation was found between the expression of OX40/OX40L and T-cell markers, such as CD3, CD4, and CD8 expression (17, 52–54). Consistent with these results, the extensive connection between OX40/OX40L and immune markers was also found in our cohort and the public cohort. Interestingly, CD3, CD4, and CD8 were identified as predictive markers for OX40L expression status on TILs. Following ESTIMATE and CIBERSORTx analyses further demonstrated higher infiltration abundance of immune cells, especially CD4(+) and CD8(+) T cells, in the high OX40/OX40L expression group. SCLC patients with high OX40/OX40L expression showed improved outcomes might be owing to augment of CD4(+) and CD8(+) T cells, which play essential roles in antitumor effect. Tumor immune heterogeneity was found between high and low OX40/OX40L expression SCLC patients.

Some limitations in the current research should be admitted. Firstly, IHC and WES data are limited. Only 102 and 41 SCLC samples separately obtained IHC and WES data. Secondly, it is a retrospective study with limited prognostic data. And different postoperative treatment might affect our study results. In addition, we did not find the relationship between metastasis that considered by clinical imaging before surgery and prognosis in previous studies (24, 27, 56). Future study with metastatic patients is warranted to further estimate the predictive efficacy of the OX40/OX40L protein expression in SCLC.

## Conclusions

In summary, we elucidated the crucial roles of OX40 and OX40L in many respects of SCLC. The OX40-OX40L axle induced immune activation and promoted immune cell infiltration in SCLC. The expression levels of OX40 and OX40L were correlated with clinical outcomes in patients with SCLC. It is worthwhile to conduct translational and clinical researches to further validate our findings in SCLC.

## Data Availability Statement 

The data presented in the study are deposited in the **Supplemental Table 1** and the China National Genebank (CNGB, https://db.cngb.org/cnsa/), accession number CNP0002096.

## Ethics Statement

The studies involving human participants were reviewed and approved by the ethics committee of Shanghai Pulmonary Hospital, Tongji University. The patients/participants provided their written informed consent to participate in this study.

## Author Contributions

Conception and design: PC and YH. Administrative support: YH. Provision of study materials or patients: YH. Collection and assembly of data: PC, LPZ, WZ, CS, and CW. Data analysis and interpretation: PC. Manuscript writing: All authors. All authors contributed to the article and approved the submitted version.

## Funding

This study was supported in part by a grant of National Natural Science Foundation of China (81802255), Clinical research project of Shanghai Pulmonary Hospital (FKLY20010), Young Talents in Shanghai (2019 QNBJ), “Dream Tutor” Outstanding Young Talents Program (fkyq1901), Clinical Research Project of Shanghai Pulmonary Hospital (fk18005), Key Discipline in 2019 (oncology), Project of Shanghai Municipal Science and Technology Commission (Project of Municipal Science and Technology Commission), Scientific research project of Shanghai Pulmonary Hospital (fkcx1903), Shanghai Municipal Commission of Health and Family Planning (2017YQ050), Innovation Training Project of SITP of Tongji University, and key projects of leading talent (19411950300). Youth project of hospital management research fund of Shanghai Hospital Association (Q1902037).

## Conflict of Interest

The authors declare that the research was conducted in the absence of any commercial or financial relationships that could be construed as a potential conflict of interest.

## Publisher’s Note

All claims expressed in this article are solely those of the authors and do not necessarily represent those of their affiliated organizations, or those of the publisher, the editors and the reviewers. Any product that may be evaluated in this article, or claim that may be made by its manufacturer, is not guaranteed or endorsed by the publisher.
