# Supplementary material for: Immune Checkpoints OX40 and OX40L in Small-Cell Lung Cancer: Predict Prognosis and Modulate Immune Microenvironment
Source: Front Oncol. 2021 Nov 25;11:713853. doi: 10.3389/fonc.2021.713853 (PMC8652148; doi:10.3389/fonc.2021.713853)
Supplement: Supplementary file 12 [file Table_2.docx]

**Table S2. Clinical and tumor characteristics of the IHC cohort (n=102)^24-27, 56^**

| **Variables** | **No. (%)** | **Variables** | **No. (%)** |
| --- | --- | --- | --- |
| Sex |  | Metastasis* |  |
| Female | 18(17.6) | No | 98(96.1) |
| Male | 84(82.4) | Yes | 4(3.9) |
| Age, mean, years | 62.7 | Postoperative treatment^^24, 27^ |  |
| <70 | 79(77.5) | Not receive | 35 (34.3) |
| ≥70 | 23(22.5) | Chemotherapy | 40 (39.2) |
| Smoking history |  | Radiotherapy | 1 (1) |
| Non-smoker | 58(56.9) | Chemotherapy plus radiotherapy | 26 (25.5) |
| Smoker | 44(43.1) | Postoperative adjuvant chemotherapy^26, 56^ |  |
| SCLC TNM staging^#^ |  | Yes | 54 (52.9) |
| I-II | 60(58.8) | No | 48 (47.1) |
| III | 42(41.2) |  |  |
| T stage ^#^ |  |  |  |
| T1-2 | 87(85.3) |  |  |
| T3-4 | 15(14.7) |  |  |
| N stage ^#^ |  |  |  |
| N0-1 | 67(65.7) |  |  |
| N2-3 | 35(34.3) |  |  |

Abbreviation: IHC, immunohistochemistry; N, lymph node; SCLC, small cell lung cancer; T, tumor, TNM, tumor-node-metastasis.

#Pathological stage.

*Clinical stage: metastasis considered by clinical imaging before surgery.

^^^ All treatment after surgery.
