# Supplementary material for: Immune Checkpoints OX40 and OX40L in Small-Cell Lung Cancer: Predict Prognosis and Modulate Immune Microenvironment
Source: Front Oncol. 2021 Nov 25;11:713853. doi: 10.3389/fonc.2021.713853 (PMC8652148; doi:10.3389/fonc.2021.713853)
Supplement: Supplementary file 13 [file Table_3.docx]

**Table S3. Clinical and tumor characteristics of the WES cohort (n=41)^28,29^**

| **Variables** | **No. (%)** | **Variables** | **No. (%)** |
| --- | --- | --- | --- |
| Sex |  | T stage* |  |
| Female | 4(9.8) | T1-2 | 10(24.4) |
| Male | 37(90.2) | T3-4 | 31(75.6) |
| Age, mean, years | 66.7 | N stage* |  |
| <70 | 31(75.6) | N0-1 | 4(9.8) |
| ≥70 | 10(24.4) | N2-3 | 37(90.2) |
| Smoking history |  | Metastasis* |  |
| Non-smoker | 8(19.5) | No | 18(43.9) |
| Smoker | 33(80.5) | Yes | 23(56.1) |
| SCLC TNM staging* |  |  |  |
| II | 1(2.4) |  |  |
| III | 17(41.5) |  |  |
| IV | 23(56.1) |  |  |

Abbreviation: N, lymph node; SCLC, small cell lung cancer; T, tumor, TNM, tumor-node-metastasis; WES, whole exon sequencing.

*Clinical stage.
