# Supplementary material for: Immune Checkpoints OX40 and OX40L in Small-Cell Lung Cancer: Predict Prognosis and Modulate Immune Microenvironment
Source: Front Oncol. 2021 Nov 25;11:713853. doi: 10.3389/fonc.2021.713853 (PMC8652148; doi:10.3389/fonc.2021.713853)
Supplement: Supplementary file 14 [file Table_4.docx]

**Table S4. Relationship between OX40/0X40L expression and clinicopathological factors**

|  | | **OX40 expression on TCs** | | | **OX40 expression on TILs** | | | **OX40L expression on TCs** | | | **OX40L expression on TILs** | | |
| --- | --- | --- | --- | --- | --- | --- | --- | --- | --- | --- | --- | --- | --- |
| **Variables** | | **Negative** | **Positive** | **P** | **Negative** | **Positive** | **P** | **Negative** | **Positive** | **P** | **Negative** | **Positive** | **P** |
| **Sex** | Female | 16 | 2 | 0.932 | 6 | 12 | 0.538 | 17 | 1 | 0.783 | 14 | 4 | 0.941 |
|  | Male | 78 | 6 |  | 22 | 62 |  | 83 | 1 |  | 66 | 18 |  |
| **Age** | <70 | 73 | 6 | 1 | 21 | 58 | 0.716 | 79 | 0 | **0.049** | 61 | 18 | 0.791 |
|  | ≥70 | 21 | 2 |  | 7 | 16 |  | 21 | 2 |  | 19 | 4 |  |
| **Smoker** | No | 52 | 6 | 0.479 | 19 | 39 | 0.168 | 56 | 2 | 0.505 | 47 | 11 | 0.463 |
|  | Yes | 42 | 2 |  | 9 | 35 |  | 44 | 0 |  | 33 | 11 |  |
| **SCLC staging** | Stage I-II | 55 | 5 | 1 | 12 | 48 | **0.044** | 59 | 1 | 1 | 48 | 12 | 0.645 |
|  | Stage III | 39 | 3 |  | 16 | 26 |  | 41 | 1 |  | 32 | 10 |  |
| **Synaptophysin** | Negative | 10 | 1 | 1 | 7 | 4 | **0.009** | 11 | 0 | 1 | 10 | 1 | 0.26 |
|  | Positive | 81 | 6 |  | 19 | 68 |  | 85 | 2 |  | 66 | 21 |  |
| **P40** | Negative | 77 | 5 | 0.508 | 18 | 64 | **0.013** | 81 | 1 | 1 | 63 | 19 | 1 |
|  | Positive | 2 | 1 |  | 3 | 0 |  | 3 | 0 |  | 3 | 0 |  |
| **TTF-1** | Negative | 18 | 1 | 1 | 6 | 13 | 0.441 | 18 | 1 | 0.845 | 12 | 7 | 0.1 |
|  | Positive | 72 | 6 |  | 18 | 60 |  | 77 | 1 |  | 63 | 15 |  |
| **CD56** | Negative | 5 | 0 | 1 | 1 | 4 | 1 | 4 | 1 | 0.229 | 4 | 1 | 1 |
|  | Positive | 77 | 7 |  | 23 | 61 |  | 83 | 1 |  | 65 | 19 |  |
| **Chromogranin A** | Negative | 40 | 2 | 0.638 | 11 | 31 | 0.819 | 41 | 1 | 1 | 32 | 10 | 0.893 |
|  | Positive | 48 | 5 |  | 15 | 38 |  | 52 | 1 |  | 41 | 12 |  |
| **KI67** | Negative | 1 | 0 | 1 | 0 | 1 | 1 | 1 | 0 | 1 | 0 | 1 | 0.227 |
|  | Positive | 59 | 6 |  | 17 | 48 |  | 63 | 2 |  | 51 | 14 |  |
| **CK5/6** | Negative | 73 | 6 | 1 | 22 | 57 | 1 | 77 | 2 | 1 | 61 | 18 | 1 |
|  | Positive | 2 | 0 |  | 1 | 1 |  | 2 | 0 |  | 2 | 0 |  |
| **P63** | Negative | 26 | 3 | 0.662 | 9 | 19 | 0.767 | 27 | 1 | 1 | 20 | 8 | 0.38 |
|  | Positive | 11 | 0 |  | 3 | 8 |  | 11 | 0 |  | 10 | 1 |  |

Abbreviation: CK5/6, cytokeratin5/6; SCLC, small cell lung cancer; TCs, tumor cells; TILs, tumor infiltrating lymphocytes; TTF-1, Thyroid transcription factor-1; OX40L, OX40 ligand; P, P value for whole.

Statistically significant data were marked with bold and underline.
