# Supplementary material for: Immune Checkpoints OX40 and OX40L in Small-Cell Lung Cancer: Predict Prognosis and Modulate Immune Microenvironment
Source: Front Oncol. 2021 Nov 25;11:713853. doi: 10.3389/fonc.2021.713853 (PMC8652148; doi:10.3389/fonc.2021.713853)
Supplement: Supplementary file 15 [file Table_5.docx]

**Table S5. Relationship between OX40/0X40L expression and other immune markers**

|  | **OX40 on TCs** | | **OX40 on TILs** | | **OX40L on TCs** | | **OX40L on TILs** | |
| --- | --- | --- | --- | --- | --- | --- | --- | --- |
| **Variables** | **R** | **P** | **R** | **P** | **R** | **P** | **R** | **P** |
| **OX40 on TCs** | 1 | **<0.001** | -0.065 | 0.518 | 0.319 | **0.001** | -0.094 | 0.349 |
| **OX40 on TCs** | -0.065 | 0.518 | 1 | **<0.001** | -0.125 | 0.211 | 0.196 | **0.048** |
| **OX40L on TCs** | 0.319 | **0.001** | -0.125 | 0.211 | 1 | **<0.001** | 0.166 | 0.096 |
| **OX40L on TILs** | -0.094 | 0.349 | 0.196 | **0.048** | 0.166 | 0.096 | 1 | **<0.001** |
| **PD-1 on TILs** | -0.062 | 0.539 | 0.291 | **0.003** | -0.107 | 0.283 | 0.314 | **0.001** |
| **PD-L1 on TCs** | -0.043 | 0.668 | 0.11 | 0.269 | -0.037 | 0.712 | 0.274 | **0.005** |
| **PD-L1 on TILs** | -0.13 | 0.192 | 0.232 | **0.019** | -0.098 | 0.329 | 0.267 | **0.007** |
| **CD3** | -0.148 | 0.138 | 0.397 | **<0.001** | -0.113 | 0.26 | 0.432 | **<0.001** |
| **CD4** | -0.091 | 0.364 | 0.331 | **0.001** | -0.074 | 0.462 | 0.461 | **<0.001** |
| **CD8** | -0.117 | 0.24 | 0.224 | **0.023** | -0.1 | 0.319 | 0.32 | **0.001** |
| **FOXP3** | -0.131 | 0.19 | 0.056 | 0.579 | -0.073 | 0.464 | 0.291 | **0.003** |

Abbreviation: SCLC, small cell lung cancer; PD-1, program death-1; PD-L1, program death-ligand 1; R, correlation coefficient; TCs, tumor cells; TILs, tumor infiltrating lymphocytes; OX40L, OX40 ligand; P, P value for whole.

Statistically significant data were marked with bold and underline.
