# Supplementary material for: Immune Checkpoints OX40 and OX40L in Small-Cell Lung Cancer: Predict Prognosis and Modulate Immune Microenvironment
Source: Front Oncol. 2021 Nov 25;11:713853. doi: 10.3389/fonc.2021.713853 (PMC8652148; doi:10.3389/fonc.2021.713853)
Supplement: Supplementary file 16 [file Table_6.docx]

**Table S6. Logistic regression for OX40 expression on TILs**

|  | **Univariate** | | | **Multivariate** | | |
| --- | --- | --- | --- | --- | --- | --- |
| **Variables** | **OR** | **95%CI** | **P** | **OR** | **95%CI** | **P** |
| **Gender (Female vs. Male)** | 1.409 | 0.472-4.208 | 0.538 |  |  |  |
| **Age (<70 vs. ≥70)** | 0.828 | 0.299-2.292 | 0.716 |  |  |  |
| **Smoking status (Non-smoker vs. Smoker)** | 1.895 | 0.759-4.730 | 0.171 |  |  |  |
| **SCLC staging (I-II vs. III)** | 0.406 | 0.167-0.987 | **0.047** | 0.467 | 0.174-1.255 | 0.467 |
| **OX40 on TCs (negative vs. positive)** | 0.343 | 0.080-1.478 | 0.151 |  |  |  |
| **OX40L on TCs (negative vs. positive)** | 0.370 | 0.022-6.123 | 0.487 |  |  |  |
| **OX40L on TILs (negative vs. positive)** | 4.815 | 1.045-22.169 | **0.044** | 2.425 | 0.428-13.740 | 0.317 |
| **PD-1 on TILs (negative vs. positive)** | 3.291 | 1.197-9.048 | **0.021** | 1.570 | 0.462-5.335 | 0.470 |
| **PD-L1 on TILs (negative vs. positive)** | 3.702 | 1.270-10.797 | **0.017** | 1.960 | 0.462-5.335 | 0.333 |
| **CD3 (negative vs. positive)** | 4.107 | 1.596-10.567 | **0.003** | 1.508 | 0.414-5.493 | 0.533 |
| **CD4 (negative vs. positive)** | 7.479 | 2.076-26.940 | **0.002** | 4.671 | 0.846-25.778 | 0.077 |
| **CD8 (negative vs. positive)** | 3.867 | 1.215-12.295 | **0.022** | 1.215 | 0.229-6.448 | 0.819 |
| **FOXP3 (negative vs. positive)** | 3.136 | 1.073-9.168 | **0.037** | 0.386 | 0.062-2.393 | 0.307 |

Abbreviation: TCs, tumor cells; TILs, tumor infiltrating lymphocytes; PD-1, program death-1; PD-L1, program death-ligand 1; FOXP3, forkhead box protein P3; OX40L, OX40 ligand; OR, Odds Ratio; P, P value for whole; 95% CI, 95% confidence interval. Statistically significant data were marked with bold and underline.
