# Supplementary material for: Immune Checkpoints OX40 and OX40L in Small-Cell Lung Cancer: Predict Prognosis and Modulate Immune Microenvironment
Source: Front Oncol. 2021 Nov 25;11:713853. doi: 10.3389/fonc.2021.713853 (PMC8652148; doi:10.3389/fonc.2021.713853)
Supplement: Supplementary file 17 [file Table_7.docx]

**Table S7. Logistic regression for OX40L expression on TILs**

|  | **Univariate** | | | **Multivariate** | | |
| --- | --- | --- | --- | --- | --- | --- |
| **Variables** | **OR** | **95%CI** | **P** | **OR** | **95%CI** | **P** |
| **Gender (Female vs. Male)** | 0.955 | 0.280-3.257 | 0.941 |  |  |  |
| **Age (<70 vs. ≥70)** | 0.713 | 0.215-2.368 | 0.581 |  |  |  |
| **Smoking status (Non-smoker vs. Smoker)** | 1.424 | 0.553-3.671 | 0.464 |  |  |  |
| **SCLC staging (I-II vs. III)** | 1.25 | 0.483-3.235 | 0.646 |  |  |  |
| **OX40 on TCs (negative vs. positive)** | 1.233 | 0.231-6.583 | 0.806 |  |  |  |
| **OX40 on TILs (negative vs. positive)** | 4.815 | 1.046-22.169 | **0.044** | 1.991 | 0.351-11.281 | 0.436 |
| **OX40L on TCs (negative vs. positive)** | 3.762 | 0.226-62.289 | 0.356 |  |  |  |
| **PD-1 on TILs (negative vs. positive)** | 4.451 | 1.618-12.242 | **0.004** | 1.813 | 0.523-6.283 | 0.348 |
| **PD-L1 on TILs (negative vs. positive)** | 5.311 | 1.916-14.719 | **0.001** | 2.075 | 0.568-7.578 | 0.269 |
| **PD-L1 on TCs (negative vs. positive)** | 7.9 | 0.682-91.555 | 0.098 |  |  |  |
| **CD3 (negative vs. positive)** | 13.529 | 2.960-61.837 | **0.001** | 5.515 | 0.900-33.805 | 0.065 |
| **CD4 (negative vs. positive)** | 7.030 | 2.438-20.269 | **<0.001** | 1.683 | 0.400-7.160 | 0.475 |
| **CD8 (negative vs. positive)** | 4.333 | 1.612-11.652 | **0.004** | 0.913 | 0.214-3.889 | 0.902 |
| **FOXP3 (negative vs. positive)** | 4.917 | 1.806-13.382 | **0.002** | 0.930 | 0.184-4.699 | 0.930 |

Abbreviation: TCs, tumor cells; TILs, tumor infiltrating lymphocytes; PD-1, program death-1; PD-L1, program death-ligand 1; FOXP3, forkhead box protein P3; OX40L, OX40 ligand; OR, Odds Ratio; P, P value for whole; 95% CI, 95% confidence interval. Statistically significant data were marked with bold and underline.
