# Supplementary material for: Immune Checkpoints OX40 and OX40L in Small-Cell Lung Cancer: Predict Prognosis and Modulate Immune Microenvironment
Source: Front Oncol. 2021 Nov 25;11:713853. doi: 10.3389/fonc.2021.713853 (PMC8652148; doi:10.3389/fonc.2021.713853)
Supplement: Supplementary file 18 [file Table_8.docx]

**Table S8. The top 10 Gene Ontology (GO) terms of each category between the high and low OX40 expression groups.**

| **Categories** | **GO ID** | **Go terms** | **P value** |
| --- | --- | --- | --- |
| **Molecular function** | GO:0019955 | cytokine binding | 3.51E-15 |
|  | GO:0140375 | immune receptor activity | 2.24E-10 |
|  | GO:0004896 | cytokine receptor activity | 2.87E-09 |
|  | GO:0005126 | cytokine receptor binding | 4.79E-09 |
|  | GO:0019838 | growth factor binding | 2.32E-08 |
|  | GO:0005178 | integrin binding | 3.20E-08 |
|  | GO:0042379 | chemokine receptor binding | 5.07E-08 |
|  | GO:0005201 | extracellular matrix structural constituent | 6.04E-08 |
|  | GO:0005125 | cytokine activity | 1.40E-07 |
|  | GO:0048020 | CCR chemokine receptor binding | 1.71E-07 |
| **Cellular components** | GO:0030667 | secretory granule membrane | 1.79E-21 |
|  | GO:0070820 | tertiary granule | 7.16E-20 |
|  | GO:0009897 | external side of plasma membrane | 2.54E-19 |
|  | GO:0062023 | collagen-containing extracellular matrix | 7.63E-18 |
|  | GO:0045335 | phagocytic vesicle | 2.64E-17 |
|  | GO:0060205 | cytoplasmic vesicle lumen | 3.20E-16 |
|  | GO:0031983 | vesicle lumen | 4.52E-16 |
|  | GO:0034774 | secretory granule lumen | 1.96E-15 |
|  | GO:0042581 | specific granule | 1.48E-14 |
|  | GO:0005766 | primary lysosome | 4.46E-13 |
| **Biological processes** | GO:0042119 | neutrophil activation | 2.16E-39 |
|  | GO:0002446 | neutrophil mediated immunity | 2.76E-39 |
|  | GO:0042110 | T cell activation | 5.92E-39 |
|  | GO:0002283 | neutrophil activation involved in immune response | 2.01E-38 |
|  | GO:0043312 | neutrophil degranulation | 4.57E-38 |
|  | GO:0007159 | leukocyte cell-cell adhesion | 4.30E-33 |
|  | GO:0034341 | response to interferon-gamma | 4.75E-33 |
|  | GO:0051249 | regulation of lymphocyte activation | 3.16E-32 |
|  | GO:0070661 | leukocyte proliferation | 4.50E-32 |
|  | GO:0045785 | positive regulation of cell adhesion | 1.32E-30 |
