# Supplementary material for: Immune Checkpoints OX40 and OX40L in Small-Cell Lung Cancer: Predict Prognosis and Modulate Immune Microenvironment
Source: Front Oncol. 2021 Nov 25;11:713853. doi: 10.3389/fonc.2021.713853 (PMC8652148; doi:10.3389/fonc.2021.713853)
Supplement: Supplementary file 19 [file Table_9.docx]

**Table S9.** **The top 10 KEGG enriched pathways of DEGs between the high and low OX40 expression groups.**

| **KEGG ID** | **KEGG enriched pathways** | **P value*** |
| --- | --- | --- |
| hsa04060 | Cytokine-cytokine receptor interaction | 5.66E-15 |
| hsa04062 | Chemokine signaling pathway | 5.70E-13 |
| hsa04621 | NOD-like receptor signaling pathway | 1.12E-10 |
| hsa04380 | Osteoclast differentiation | 2.49E-16 |
| hsa04142 | Lysosome | 7.17E-15 |
| hsa05164 | Influenza A | 7.02E-10 |
| hsa04061 | Viral protein interaction with cytokine and cytokine receptor | 3.06E-13 |
| hsa04064 | NF-kappa B signaling pathway | 1.49E-10 |
| hsa05142 | Chagas disease | 1.68E-09 |
| hsa05340 | Primary immunodeficiency | 4.45E-11 |

Abbreviation: *****, P values were calculated by hypergeometric test; DEGs, differentially expressed genes; KEGG, Kyoto Encyclopedia of Genes and Genomes.
