# Supplementary material for: Immune Checkpoints OX40 and OX40L in Small-Cell Lung Cancer: Predict Prognosis and Modulate Immune Microenvironment
Source: Front Oncol. 2021 Nov 25;11:713853. doi: 10.3389/fonc.2021.713853 (PMC8652148; doi:10.3389/fonc.2021.713853)
Supplement: Supplementary file 20 [file Table_10.docx]

**Table S10. The top 10 Gene Ontology (GO) terms of each category between the high and low OX40L expression groups.**

| **Categories** | **Go ID** | **Go terms** | **P value** |
| --- | --- | --- | --- |
| **Molecular function** | GO:0005125 | cytokine activity | 1.60E-09 |
|  | GO:0005126 | cytokine receptor binding | 3.98E-08 |
|  | GO:0019955 | cytokine binding | 5.80E-08 |
|  | GO:0140375 | immune receptor activity | 5.80E-08 |
|  | GO:0051015 | actin filament binding | 6.11E-08 |
|  | GO:0005178 | integrin binding | 9.59E-08 |
|  | GO:0043394 | proteoglycan binding | 2.04E-07 |
|  | GO:0003779 | actin binding | 5.81E-07 |
|  | GO:0004896 | cytokine receptor activity | 7.33E-07 |
|  | GO:0035325 | Toll-like receptor binding | 1.12E-06 |
| **Cellular components** | GO:0030667 | secretory granule membrane | 3.87E-20 |
|  | GO:0034774 | secretory granule lumen | 2.73E-17 |
|  | GO:0060205 | cytoplasmic vesicle lumen | 4.42E-17 |
|  | GO:0031983 | vesicle lumen | 5.61E-17 |
|  | GO:0070820 | tertiary granule | 4.46E-15 |
|  | GO:0005775 | vacuolar lumen | 1.69E-14 |
|  | GO:0005766 | primary lysosome | 5.13E-14 |
|  | GO:0042582 | azurophil granule | 5.13E-14 |
|  | GO:0009897 | external side of plasma membrane | 5.59E-14 |
|  | GO:0042581 | specific granule | 8.01E-13 |
| **Biological processes** | GO:0042110 | T cell activation | 2.51E-42 |
|  | GO:0042119 | neutrophil activation | 1.14E-40 |
|  | GO:0043312 | neutrophil degranulation | 7.43E-40 |
|  | GO:0002283 | neutrophil activation involved in immune response | 1.22E-39 |
|  | GO:0002446 | neutrophil mediated immunity | 7.37E-39 |
|  | GO:0051249 | regulation of lymphocyte activation | 2.10E-36 |
|  | GO:0007159 | leukocyte cell-cell adhesion | 3.58E-35 |
|  | GO:0050863 | regulation of T cell activation | 2.12E-33 |
|  | GO:0070661 | leukocyte proliferation | 6.48E-33 |
|  | GO:0070663 | regulation of leukocyte proliferation | 2.12E-31 |
