# Supplementary material for: Immune Checkpoints OX40 and OX40L in Small-Cell Lung Cancer: Predict Prognosis and Modulate Immune Microenvironment
Source: Front Oncol. 2021 Nov 25;11:713853. doi: 10.3389/fonc.2021.713853 (PMC8652148; doi:10.3389/fonc.2021.713853)
Supplement: Supplementary file 21 [file Table_11.docx]

**Table S11. The top 10 KEGG enriched pathways of DEGs between the high and low OX40L expression groups.**

| **KEGG ID** | **KEGG enriched pathways** | **P value*** |
| --- | --- | --- |
| hsa04060 | Cytokine-cytokine receptor interaction | 3.37E-11 |
| hsa05152 | Tuberculosis | 2.01E-10 |
| hsa04062 | Chemokine signaling pathway | 1.02E-09 |
| hsa04380 | Osteoclast differentiation | 9.23E-13 |
| hsa04621 | NOD-like receptor signaling pathway | 4.75E-09 |
| hsa04142 | Lysosome | 6.39E-09 |
| hsa04061 | Viral protein interaction with cytokine and cytokine receptor | 9.48E-09 |
| hsa05133 | Pertussis | 2.15E-08 |
| hsa05140 | Leishmaniasis | 2.65E-08 |
| hsa05340 | Primary immunodeficiency | 3.78E-09 |

Abbreviation: *****, P values were calculated by hypergeometric test; DEGs, differentially expressed genes; KEGG, Kyoto Encyclopedia of Genes and Genomes.
